# Association between triglyceride-glucose index and sarcopenic obesity in adults: a population-based study

**DOI:** 10.3389/fnut.2025.1452512

**Published:** 2025-04-11

**Authors:** Zhengmei Zhao, Ruiting Cai, Lili Tao, Yuxiao Sun, Keping Sun

**Affiliations:** ^1^Department of Geriatrics, Shanghai East Hospital, School of Medicine, Tongji University, Shanghai, China; ^2^Department of Cardiology, Shanghai East Hospital, School of Medicine, Tongji University, Shanghai, China

**Keywords:** sarcopenic obesity, triglyceride-glucose index, insulin resistance, population-based study, CHARLS database

## Abstract

**Background:**

Sarcopenic obesity is characterized by the coexistence of sarcopenia and obesity, which has been demonstrated to be linked to insulin resistance. The triglyceride-glucose (TyG) index is a novel surrogate indicator of insulin resistance. However, the relationship between sarcopenic obesity and the TyG index remains unclear. This population-based study aimed to investigate the association between TyG index and sarcopenic obesity in adults.

**Methods:**

This study utilized data from the China Health and Retirement Longitudinal Study (CHARLS), individuals aged over 45 years were categorized into sarcopenia, obesity, sarcopenic obesity, and control groups. Baseline characteristics were analyzed across these groups. Logistic regression models were employed to explore the association between the TyG index and sarcopenic obesity, adjusting for confounding variables. Subgroup analyses were conducted to explore the potential variations in this association across different demographic and clinical variables.

**Results:**

A total of 9,485 individuals were included in the study, with the sarcopenic obesity group exhibiting a higher TyG index (8.96 ± 0.61) compared to other groups (*p* < 0.001). A significant and independent association was observed between higher TyG index and increased likelihood of sarcopenic obesity, even after adjusting for confounders. Participants in the highest TyG index tertile had a 1.82-fold increase in the risk of sarcopenic obesity compared to those in the lowest tertile. Subgroup analyses revealed significant associations between TyG index and sarcopenic obesity across various demographic and clinical variables, particularly in individuals aged over 60 years, females, and those with chronic diseases.

**Conclusion:**

The findings suggest a strong association between elevated TyG index and sarcopenic obesity in adults, independent of demographic and clinical factors. Further large-scale prospective studies are warranted to validate and expand upon these findings.

## Introduction

Sarcopenic obesity, characterized by the coexistence of low muscle mass and high adiposity, has emerged as a significant public health concern in the contemporary era (1). With global populations aging, sarcopenic obesity has garnered increasing attention due to its association with adverse health outcomes, including impaired physical function (2), diminished quality of life (3), increased risk of chronic diseases (4, 5), and heightened mortality rates (6). However, despite its recognized importance, the prevalence of sarcopenic obesity varies notably due to challenges in establishing a precise and universally accepted definition of sarcopenic obesity worldwide (7). A meta-analysis encompassing 50 studies revealed a global prevalence of 11% of sarcopenic obesity among adults aged 60 years (8). Similarly, another meta-analysis including 106 clinical studies with 167,151 elderlies demonstrated an estimated prevalence of 9% in both male and female elderly populations (9). The prevalence of sarcopenic obesity is on the rise with age, as evidenced by a cohort study which included individuals aged 18 to 90 years, with a prevalence of 1.4% in women and 0.9% in men, markedly rising to 16.7% in the 80–89 years age group (10). This trend is concerning, particularly considering the compounded effects of sarcopenia and obesity.

The pathophysiology of sarcopenic obesity is complicated, influenced by several factors, including aging-related alternations in body composition, sex-specific hormonal changes, chronic inflammation, collectively leading to intramyocellular lipids deposition and reduced muscle mass (1, 11). Notably, insulin resistance plays a pivotal role in the development of sarcopenic obesity. Insulin resistance is a condition in which cells become less responsive to the effects of insulin, leading to impaired glucose uptake and utilization. The dysregulation of insulin signaling has been elucidated to disrupt protein anabolism and induce muscle atrophy (12). Studies have demonstrated the detrimental impact of insulin resistance on muscle catabolism, leading to progressive declines in muscle strength and quality associated with metabolic dysfunction (13).

The Triglyceride-glucose (TyG) index, serving as a surrogate marker of insulin resistance derived from levels of both fasting triglyceride and glucose, has been identified as a potential indicator of metabolic dysfunction (14). Accumulating evidence has established associations between the TyG index and multiple health conditions, including metabolic syndrome (15), type 2 diabetes (16), non-alcoholic fatty liver disease (17), and cardiovascular events (18). In addition to these conditions, research has also linked the TyG index to sarcopenia. A cross-sectional study involving 4,030 participants aged 20 years and above reported that a 1-unit increase in the TyG index was associated with a 31% higher likelihood of sarcopenia (19). Another study explored the relationship between the TyG index and the components of sarcopenia, including low muscle mass and low muscle strength. The findings revealed a higher TyG index was associated with increases odds of own muscle mass and low muscle strength, suggesting the TyG index might serve as a valuable screening tool for early identification of individuals at risk of sarcopenia (20). However, the association between the TyG index and sarcopenic obesity remains underexplored. Sarcopenic obesity poses unique metabolic challenges, as the coexistence of muscle loss and increased adiposity may further exacerbate insulin resistance, which in turn influence the TyG index. Considering the intricate interplay between metabolic dysfunction and body compositions alternations observed in sarcopenic obesity, further studies are essential to elucidate the potential role and associations of TyG index for sarcopenia obesity.

The China Health and Retirement Longitudinal Study (CHARLS), a nationally representative survey, provides a robust platform for investigating epidemiological trends and associations in health-related outcomes (21). Utilizing the comprehensive data from CHARLS, we aim to explore the association between the TyG index and sarcopenic obesity among individuals aged over 45. By elucidating the relationship between TyG index and sarcopenic obesity, this study may contribute to a deeper understanding of the underlying mechanisms linking insulin resistance and body composition.

## Methods

### Data and sample sources

**Figure 1:**
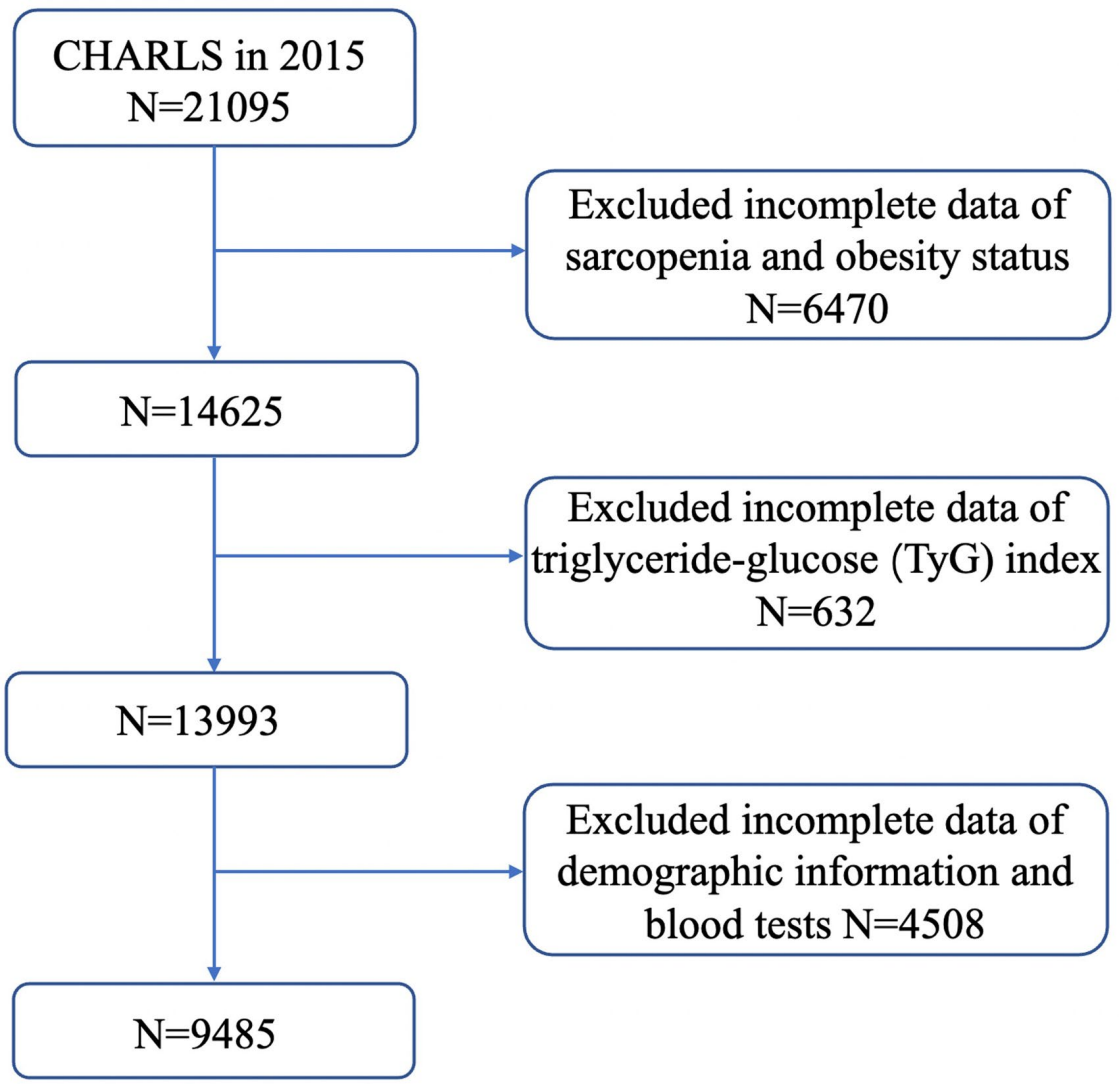
Flowchart of the study.

The data for this study were collected from the China Health and Retirement Longitudinal Study (CHARLS) in 2015, a nationally representative survey on community-based population, conducted by the China Center for Economic Research at Peking University. The CHARLS database was established in 2011 and recruited participants through utilizing multistage stratified probability sampling procedure. The sample covered 150 counties, and 450 villages/urban communities, involving 17,708 individuals nationwide. The CHARLS is an ongoing survey with exams performed every 2 to 3 years for a total of 4 waves. The demographic characteristics, including age, sex, health status, medical care, anthropometric measurements, as well as blood sample collection were included in the surveys. A more detailed CHARLS survey design has been published in previous literature (21). The CHARLS datasets can be accessed on its official website.^1^ The CHARLS protocol was approved by the Biomedical Ethics Committee of Peking University and all participants were required to provide informed consent. In this study, we retrospectively analyzed data from CHARLS 2015 and exclude 11,610 individuals due to (1) missing data of sarcopenia and obesity status; (2) missing data of TyG index; (3) missing data of demographic information and blood test. The cross-sectional analysis included 9,845 individuals. The detailed flowchart of the study is presented in Figure 1.

### Assessment of sarcopenia obesity

In this study, sarcopenia was defined using the recommended diagnostic algorithm of the Asian Working Group for Sarcopenia (AWGS) 2019 (22). Individuals with a low appendicular skeletal muscle mass (ASM) or both a low handgrip strength and a low gait speed were considered to have sarcopenia. ASM was calculated according to a published physical measurement formula verified among the Chinese population (23, 24). The equation of ASM was shown as follows: ASM = 0.193 × Weight + 0.107 × Height − 4.157 × sex (men = 1, women = 2) − 0.037 × age − 2.631. The appendicular muscle mass index (ASMI) was calculated using the ASM divided by the square of the height. The cut-off values of the ASMI were based on the sex-specific 20% lowest percentile of the study population (25). Therefore, a low muscle mass was classified as ASMI less than 7.0 kg/m^2^ in men and 5.34 kg/m^2^ in women in this study. Handgrip strength was measured with a Yuejian™ WL-1000 dynamometer (Nantong Yuejian Physical Measurement Instrument Co., LTD., Nantong, China) (21). Subjects were in standing position, and then hold the dynamometer with dominant or nondominant hand for a few seconds. The handgrip strength was measured twice for each hand with maximum effort. In this study, we calculated the maximum grip strength and then took the average of the available values. According to the guideline of AWGS 2019, the cut-off points for low handgrip strength are recommended as <28 kg in men and <18 kg in women (22). For the gait speed, participants were instructed to walk over 2.5 m course at usual pace twice, and the average value of gait speed was used for analysis. The slow walking speed was defined as <1.0 m/s according to the AWGS 2019 criteria (22). Obesity is defined as body mass index (BMI) ≥ 25 kg/m^2^ according to WHO standard for Asian populations (26), which was calculated as weight in kilogram divided by height squared in meter. In this study, participants were classified into four body composition categories: (1) sarcopenia: met criteria for sarcopenia but not obesity; (2) obesity: met criteria for obesity but not sarcopenia; (3) sarcopenic obesity: met criteria for sarcopenia and obesity; and (4) non-sarcopenic non-obese (control group): did not meet definition for obesity or sarcopenia.

### Definition of TyG index

The venous blood samples were collected from each participant following overnight fasting. The fresh blood samples were immediately processed and then stored at −20°C, and transported to the Chinese CDC in Beijing within 2 weeks until the assay was analyzed. The fasting glucose, total cholesterol, high-density lipoprotein cholesterol, low-density lipoprotein cholesterol, triglyceride was detected by the Enzymatic colormetric test. The Glycosylated hemoglobin (HbA1c) was measured by Boronate affinity HPLC. TyG index was calculated as in (fasting triglyceride [mg/dl] × fasting glucose [mg/dl]/^2^) (27).

### Potential covariates

Covariates comprised age, gender, marital status, education, smoking status, drinking status, BMI, ASMI, systolic blood pressure (SBP), diastolic blood pressure (DBP), hypertension, dyslipidemia, diabetes, heart disease, chronic lung disease, cancer, and stroke. Education level was classified as illiteracy, primary school, middle school, high school or above. Marital status was allocated into married, separated/divorced/widowed, unmarried. Heath behavior related factors included smoking and drinking status, were defined as former or current smoking/drinking (yes) and never smoking/drinking (no) groups. Hypertension was determined by self-reported doctor diagnosis and baseline blood pressure, and SBP of > = 140 mmHg or DBP of > = 90 mmHg was defined as hypertension. Dyslipidemia was identified by self-reported diagnosis and baseline lipid level (total cholesterol > = 240 mg/dl, LDL-C > 160 mg/dl, triglycerides > = 200 mg/dl or HDL-C < 40 mg/dl). Other comorbid conditions including heart disease, chronic lung disease, cancer, and stroke, were determined by the self-reported conditions in the questionnaire.

### Statistical analysis

Continuous variables were described as mean with standard deviation (SD), and categorical data were presented as numbers and percentages. One-way analysis of variance (ANOVA) or Kruskal-Wallis test were used to compare the differences for continuous data, whereas Pearson chi-square test or Fisher’s exact test for count data. Logistic regression models were used to explore the association between TyG index and sarcopenia obesity in different models. The variance inflation factor (VIF) was implemented to determine the collinearity of independent variables. Independent variables with VIF greater than 10 were excluded. In model 1, no covariates were adjusted. Model 2 was adjusted for age, gender, education levels, marital status, smoking and drinking status. In model 3, age, gender, education levels, marital status, smoking, drinking, fasting glucose, glycated hemoglobin, serum creatinine, handgrip strength, walking speed, and chronic medical conditions were adjusted. Age (<60/≥60 years), gender (female/male), hypertension (yes/no), dyslipidemia (yes/no), diabetes (yes/no), cancer (yes/no), heart disease (yes/no), stroke (yes/no) were stratified for subgroup analysis. R version 4.2.0^2^ were utilized for all analyses. Statistically significance was defined as *p* value <0.05.

## Results

### Characteristics of study population

The baseline characteristics are presented in Table 1. Overall, a total of 9,485 individuals were included in the study, with an average age of 62.36 ± 9.03 years, including 46.58% male. Of all the participants, 3,778 (39.83%) were categorized into the sarcopenia group, 1766 (18.62%) into the obesity group, 1,586 (16.72%) into the sarcopenic obesity group, and 2,355 (24.83%) into control group. We compared demographic and clinical indicators for participants across these groups. Notably, the individuals of sarcopenic obesity group were older and exhibited significantly higher proportion of females compared to the other groups (*p* < 0.001). Differences in the sociodemographic factors, health behavior related indicators, and blood biomarkers were also statistically significant across the four groups (*p* < 0.001). The prevalence of hypertension, diabetes, cancer, heart diseases, and stroke were notably elevated in the sarcopenic obesity group. Moreover, hand grip strength was significantly lower in sarcopenic obesity group (20.97 ± 4.77) kg compared to the other groups (*p* < 0.001), while the TyG index was higher in the sarcopenic obesity group (8.96 ± 0.61) (*p* < 0.001).

**Table 1:** Characteristics of study population.

Variables	Overall *n* = 9,485	Sarcopenia *n* = 3,778	Obesity *n* = 1766	Sarcopenic obesity *n* = 1,586	Control *n* = 2,355	*p*-value
Age, years	62.36 ± 9.03	71.44 ± 9.45	61.38 ± 8.58	75.04 ± 8.04	61.90 ± 8.40	<0.001
Sex, *n* (%)						<0.001
Male	4,418(46.58)	1,276(33.78)	1,115(63.14)	196(12.36)	1831(77.75)	
Female	5,067(53.42)	2,502(66.22)	651(36.86)	1,390(87.64)	524(22.25)	
Education, *n* (%)						<0.001
Illiteracy	4,339(45.75)	2,192(58.02)	518(29.33)	910(57.38)	719(30.53)	
Primary school	2,198(23.17)	809(21.41)	420(23.78)	332(20.93)	637(27.05)	
Middle school	1997(21.05)	558(14.77)	533(30.18)	248(15.64)	658(27.94)	
High school or above	951(10.03)	219(5.80)	295(16.70)	96(6.05)	341(14.48)	
Marital status, *n* (%)						<0.001
Married	8,458(89.17)	3,211(84.99)	1,678(95.02)	1,387(87.45)	2,182(92.65)	
Separated/Divorced/Widowed	960(10.12)	534(14.13)	84(4.76)	195(12.30)	147(6.24)	
Not married	67(0.71)	33(0.87)	4(0.23)	4(0.25)	26(1.10)	
Smoking, *n* (%)	8,735(92.09)	3,560(94.23)	1,552(87.88)	1,513(95.40)	2,110(89.60)	<0.001
Drinking, *n* (%)	3,137(33.07)	976(25.83)	707(40.03)	239(15.07)	1,215(51.59)	<0.001
ASM (kg)	17.05 ± 4.15	14.28 ± 3.21	20.88 ± 3.77	16.32 ± 2.83	19.11 ± 2.96	<0.001
BMI (kg/m^2^)	23.85 ± 3.70	21.11 ± 2.34	27.78 ± 2.46	27.84 ± 2.44	22.62 ± 1.44	<0.001
Waist circumference (cm)	85.47 ± 13.16	78.53 ± 11.34	95.49 ± 11.22	94.83 ± 10.31	82.69 ± 10.33	<0.001
SBP (mmHg)	131.95 ± 20.26	129.46 ± 20.66	135.57 ± 18.73	136.92 ± 20.12	131.21 ± 19.46	<0.001
DBP (mmHg)	75.10 ± 11.48	72.87 ± 11.55	79.21 ± 11.32	77.05 ± 11.09	75.75 ± 10.64	<0.001
Comorbidities, *n* (%)						
Hypertension	2,275(23.99)	689(18.24)	580(32.84)	617(38.90)	389(16.52)	<0.001
Diabetes	523(5.51)	164(4.34)	132(7.47)	128(8.07)	99(4.20)	<0.001
Dyslipidemia	896(9.45)	231(6.11)	271(15.34)	240(15.13)	154(6.54)	<0.001
Cancer	90(0.94)	37(0.98)	15(0.85)	24(1.51)	14(0.60)	0.03
Chronic lung diseases	934(9.85)	431(11.41)	130(7.36)	149(9.39)	224(9.51)	<0.001
Heart diseases	1,100(11.60)	421(11.14)	222(12.57)	277(17.47)	180(7.64)	<0.001
Stroke	170(1.79)	69(1.83)	29(1.64)	45(2.84)	27(1.15)	0.001
Blood biomarkers						
Fasting Glucose (mg/dl)	104.74 ± 35.98	101.43 ± 34.36	111.01 ± 42.22	109.48 ± 34.88	104.24 ± 35.07	<0.001
Glycated Hemoglobin (%)	6.06 ± 1.03	5.98 ± 0.98	6.21 ± 1.10	6.25 ± 1.09	5.99 ± 0.99	<0.001
Total Cholesterol (mg/dl)	184.72 ± 36.32	185.12 ± 36.79	182.34 ± 35.27	191.71 ± 36.33	180.04 ± 35.11	<0.001
Triglycerides (mg/dl)	135.99 ± 84.50	120.90 ± 73.88	162.95 ± 93.63	166.87 ± 93.01	127.64 ± 82.66	<0.001
HDL Cholesterol (mg/dl)	51.78 ± 12.17	54.63 ± 13.10	46.40 ± 9.42	49.23 ± 9.74	51.02 ± 11.65	<0.001
LDL Cholesterol (mg/dl)	103.17 ± 28.65	102.83 ± 28.82	102.72 ± 28.51	107.27 ± 29.00	101.04 ± 27.85	<0.001
Serum Creatinine (mg/dl)	0.84 ± 0.30	0.81 ± 0.27	0.91 ± 0.29	0.76 ± 0.22	0.91 ± 0.38	<0.001
Hand grip strength (kg)	28.69 ± 9.56	23.10 ± 7.07	36.12 ± 6.08	20.97 ± 4.77	35.64 ± 5.43	<0.001
Gait speed (m/s)	1.46 ± 2.11	1.44 ± 0.53	1.22 ± 0.38	1.51 ± 1.20	1.42 ± 4.40	0.008
TyG index	8.69 ± 0.63	8.56 ± 0.59	8.93 ± 0.62	8.96 ± 0.61	8.62 ± 0.62	<0.001

ASM, appendicular skeletal muscle mass; BMI, body mass index; SBP, systolic blood pressure; DBP, diastolic blood pressure; HDL, high-density lipoprotein cholesterol LDL, low-density lipoprotein cholesterol; TyG index, triglyceride-glucose index.

### Association of TyG index with sarcopenic obesity

The results indicated a robust association between higher TyG index and an elevated likelihood of sarcopenic obesity. This association was significant in unadjusted model 1 [OR (95%CI): 1.84 (1.70–2.00), *p* < 0.001], and minimally adjusted model 2 [OR (95%CI): 1.74 (1.60–1.90), *p* < 0.001], which accounted for age, gender, education level, marital status, smoking and drinking. Importantly, in the fully adjusted model 3, the association between TyG index and sarcopenic obesity remain stable [OR (95%CI): 2.08 (1.78–2.43), *p* < 0.001]. Furthermore, we transformed TyG index from a continuous variable to a categorical one. Compared to those in the lowest TyG index tertile, participants in the highest TyG index tertile demonstrated a statistically significant 1.82-fold increase in the risk of sarcopenic obesity [OR (95CI%): 2.82 (2.26–3.62), *p* < 0.0001]. Similarly, participants in the middle TyG index tertile exhibited a significant 51.0% increase in the risk of sarcopenic obesity with statistical significance [OR (95CI%): 1.51(1.21–1.88), *p* < 0.0001] (Table 2).

**Table 2:** Association between TyG index and sarcopenic obesity.

OR (95%CI), *p*-value			
Characteristic	Model 1	Model 2	Model 3
Continuous			
	1.84 (1.70, 2.00) <0.0001 ***	1.74 (1.60,1.90) <0.0001 ***	2.08 (1.78, 2.43) <0.0001 ***
Categories			
Tertile 1	Reference	Reference	Reference
Tertile 2	1.77 (1.52, 2.05) <0.0001 ***	1.51 (1.29, 1.76) <0.0001 ***	1.51 (1.21, 1.88) <0.0001 ***
Tertile 3	2.93 (2.54, 3.38) <0.0001 ***	2.51 (2.16, 2.92) <0.0001 ***	2.82 (2.26, 3.52) <0.0001 ***

OR, odds ratio; 95% CI, 95% confidence interval. Model 1: No adjustment; Model 2: Adjusted for age, gender, education, marital status, smoking and drinking; Model 3: Adjusted for age, gender, education, marital status, smoking, drinking, hypertension, dyslipidemia, diabetes, heart diseases, chronic lung diseases, stroke, cancer, fasting glucose, glycated hemoglobin, serum creatinine, handgrip strength, walking speed. ***, *p*<0.001.

In the fully adjusted model, age, gender, hypertension, dyslipidemia, fasting glucose, glycated hemoglobin and hand grip strength maintained significant associations with the likelihood of having sarcopenic obesity (Table 3). The female participants exhibited 1.34-fold higher odds of sarcopenic obesity compared with male participants, (*p* < 0.0001). Additionally, individuals without hypertension or dyslipidemia had a 54 and 32% lower likelihood of sarcopenic obesity, respectively. For each unit increase in the fasting glucose, the odds of sarcopenic obesity increased by 5% (*p* = 0.002), while per unit increase in hand grip strength, the odds of sarcopenic obesity were decreased by 12% (*p* < 0.0001).

**Table 3:** Multivariate regression model of sarcopenic obesity.

Variables	OR (95% CI)	*p*-value
TyG index	2.19 (1.86, 2.58)	<0.0001
Age (year)	1.04 (1.02, 1.05)	<0.0001
Female (verse male)	2.34 (1.79, 3.06)	<0.0001
Education (verse illiteracy)		
Primary school	1.11 (0.89, 1.37)	0.348
Middle school	1.05 (0.79, 1.40)	0.740
High school or above	0.70 (0.44, 1.13)	0.144
Marriage (verse married)		
Separated/Divorced/Widowed	0.95 (0.75, 1.20)	0.657
Not married	0.40 (0.05, 3.03)	0.374
Smoking (no verse yes)	0.78 (0.54, 1.11)	0.162
Drinking (no verse yes)	0.94 (0.74, 1.18)	0.593
Fasting Glucose (mg/dl)	1.05 (1.02, 1.09)	0.002
Glycated Hemoglobin (%)	1.05 (0.95, 1.17)	0.336
Serum Creatinine (mg/dl)	0.74 (0.51, 1.09)	0.125
Hypertension (no verse yes)	0.46 (0.38, 0.56)	<0.0001
Dyslipidemia (no verse yes)	0.68 (0.53, 0.87)	0.003
Diabetes (no verse yes)	0.90 (0.64, 1.25)	0.520
Cancer (no verse yes)	0.74 (0.35, 1.56)	0.430
Chronic lung diseases (no verse yes)	1.16 (0.88, 1.54)	0.294
Heart diseases (no verse yes)	0.90 (0.71, 1.13)	0.357
Stroke (no verse yes)	0.72 (0.43, 1.22)	0.223
Hand grip strength (kg)	0.90 (0.88, 0.91)	<0.0001
Gait speed (m/s)	1.02 (0.98, 1.06)	0.459

OR, odds ratio; 95% CI, 95% confidence interval. TyG index, triglyceride-glucose index.

### Subgroup analysis

**Figure 2:**
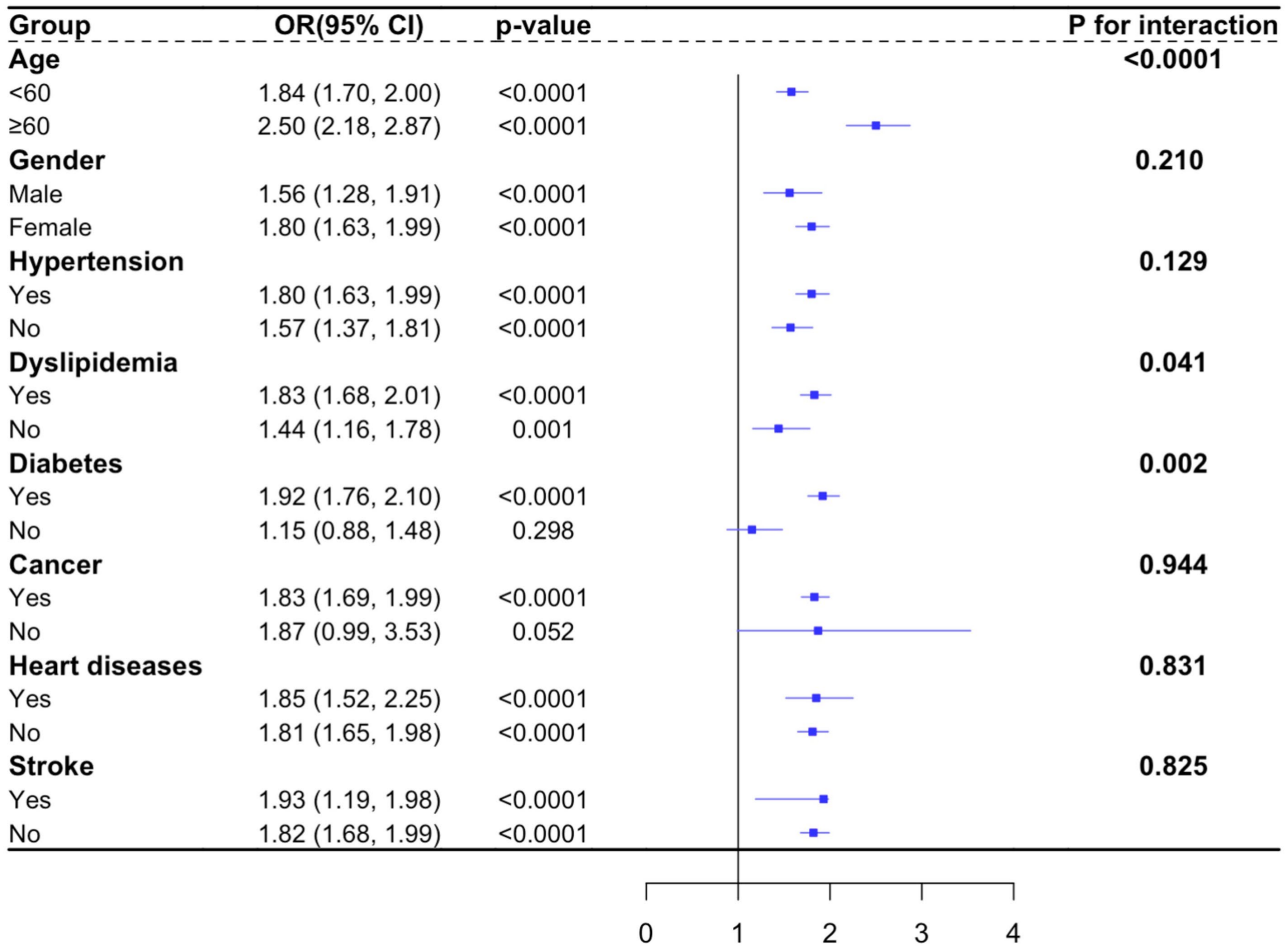
Subgroup analysis.

The findings of subgroup analysis revealed that the association between TyG index and increased odds of sarcopenic obesity varied across different subgroups (Figure 2). For the subgroup stratified by age, gender, hypertension, dyslipidemia, heart diseases and stroke, a significant relationship between TyG index and sarcopenic obesity was observed in each subgroup (all *p* < 0.05). Within subgroups stratified by diabetes and cancer, statistical significance was only evident in individuals with diabetes and cancer (*p* < 0.05). Furthermore, the interaction tests indicated significant differences among age, dyslipidemia and diabetes in the association between TyG index and sarcopenic obesity (p for interaction <0.05). The stratification of other groups, including gender, hypertension, cancer, heart diseases, and stroke, showed no significance between TyG index and sarcopenic obesity, indicating that there was no significant dependence on this positive association (p for interaction >0.05).

## Discussion

In our study with a total of 9,485 participants enrolled, we investigated the association between the TyG index and the likelihood of sarcopenic obesity in a large population sample. Our findings revealed a significant positive association between higher TyG index levels and increased odds of sarcopenic obesity, adjusting for potential confounding factors.

Consistent with previous research, our results showed that individuals with higher TyG index levels were more likely to have sarcopenic obesity, suggesting that insulin resistance, reflected by the TyG index, may play a role in the pathogenesis of this condition. This association persisted robustly even after adjustments for diverse demographic, lifestyle, and clinical variables, indicating an independent relationship between the TyG index and sarcopenic obesity. Notably, a previous cross-sectional study involving 3,821 participants aged 60 years and above, stratified into sarcopenia, obesity, and sarcopenic obesity groups, demonstrated a positive correlation between high TyG index and sarcopenic obesity, thus highlighting the utility of the TyG index as a potential indicator for sarcopenic obesity (28). To be specific, the cut-off values were ≥8.72 for men and 8.67 for women in individuals with sarcopenic obesity (28). Moreover, the relationship between sarcopenic obesity and type 2 diabetes mellitus (T2DM) has been established through a cross-sectional study involving 1,629 older adults. This study revealed that both low handgrip strength and TyG index are independently associated with T2DM (29). Similarly, homeostasis model assessment of insulin resistance (HOMA-IR) is an alternative indicator of TyG index for insulin resistance (30, 31). A population-based study explored the relationships between sarcopenic obesity and insulin resistance via HOMA-IR. The findings of that study revealed a gender-specific pattern, within the female, those with sarcopenic obesity exhibited higher HOMA-IR levels (32). This result mirrored our finding to some extent, wherein the female displayed a high risk of sarcopenic obesity compared to males. This disparity of sarcopenic obesity rates between men and women involve several factors, including sex hormones, eating behaviors, social norms (33). As the important female sex hormones, estrogen and progesterone play crucial roles in regulating metabolism and fat distribution. Deficiency in hormone levels in menopausal women promotes adipose tissue deposition and accelerate muscle loss, increasing the risk of sarcopenic obesity (34). Women are more sensitive to hedonic dietary compared to males, which may lead to sex-based variations in muscle mass, fat distribution, and metabolic health (35). In addition, societal norms and expectations regarding body image and weight may exert additional pressure on females, potentially leading to disordered eating habits that contribute to sarcopenic obesity (36).

The pathogenesis of sarcopenic obesity is multifactorial, involving intricate interactions between musculoskeletal, metabolic, hormonal, and neurological factors (37, 38). Insulin resistance plays a pivotal role in the progression of sarcopenic obesity, which impairs muscle protein synthesis and alters adipose tissue dynamics, directly leading to muscle fiber atrophy (11). In addition, insulin resistance promotes the production and secretion of pro-inflammatory adipokines, such as interleukin-6 (IL-6) and tumor necrosis factor-α (TNF-α). These cytokines contribute to chronic low-level inflammation and oxidative stress (39), exacerbating the muscle wasting and reducing muscle strength (40). Moreover, insulin resistance disrupts lipid metabolism, resulting in intramyocellular lipid accumulation, which further impairs insulin signaling and downstream metabolism. The cycle of inflammation, oxidative stress, and ectopic fat deposition promotes the progressive development of sarcopenic obesity (41).

Notably, it is crucial to recognize that sarcopenic obesity represents a complex interplay of various factors beyond insulin resistance alone. Age-related hormonal changes, including alterations in growth hormone and sex hormone levels, contribute to muscle loss and adiposity in older adults (42). Additionally, social status and health behaviors like smoking may influence the development of sarcopenic obesity (43, 44). Moreover, it has been reported that having two or more chronic diseases was prone to develop sarcopenic obesity (45). Thus, in this study, we also observed that the correlation between TyG index and sarcopenic obesity was consistent across various subgroups stratified by age, gender, hypertension, dyslipidemia, heart diseases, and stroke. However, the association was particularly pronounced in individuals with diabetes and cancer, highlighting the potential importance of managing insulin resistance in these populations to prevent sarcopenic obesity. Interestingly, our subgroup analysis also revealed significant interactions between TyG index and age, dyslipidemia, and diabetes, suggesting that the impact of TyG index on sarcopenic obesity may vary depending on these factors. Further research is needed to elucidate the underlying mechanisms and biological pathways linking TyG index, insulin resistance, and sarcopenic obesity, particularly in different subpopulations.

Strengths of our study include the large sample size and sample selection is representative. However, there are some limitations to consider. First, causality cannot be inferred in this study due to the cross-sectional data, longitudinal studies are needed to confirm our findings. Second, the stratification of other subgroups, such as education levels and marital status were not considered. Third, TyG index as an indicator for insulin resistance may not fully explain the complexity of insulin dysregulation.

## Conclusion

Our study provides evidence for an independent association between elevated TyG index and the increased likelihood of sarcopenic obesity. However, further large-scale prospective studies are warranted to validate our findings.

## Data Availability

The original contributions presented in the study are included in the article/supplementary material, further inquiries can be directed to the corresponding authors.

## Footnotes

^1^ http://charls.pku.edu.cn/en

^2^ http://www.R-project.org
